# Inter-laboratory and inter-operator reproducibility in gait analysis measurements in pediatric subjects

**DOI:** 10.1080/23335432.2019.1621205

**Published:** 2019-05-29

**Authors:** Emilia Scalona, Roberto Di Marco, Enrico Castelli, Kaat Desloovere, Marjolein Van Der Krogt, Paolo Cappa, Stefano Rossi

**Affiliations:** a Department of Mechanical and Aerospace Engineering (DIMA), “Sapienza” University of Rome, Rome, Italy; b Laboratory of Movement Analysis and Robotics (MARlab), “Bambino Gesù” Children Hospital, Passoscuro Fiumicuno (RM), Italy; c KU Leuven, Department of Rehabilitation Sciences, Laboratory for Clinical Motion Analysis University Hospital Leuven, Pellenberg, Belgium; d Department of Rehabilitation Medicine, VU University Medical Center, Amsterdam Movement Sciences, Amsterdam, The Netherlands; e Department of Economics, Engineering, Society and Business Organization (DEIM), University of Tuscia, Viterbo, Italy

**Keywords:** Gait analysis, electromyography, intra-subject repeatability, inter-operator reproducibility, inter-laboratory reproducibility, pediatric subjects

## Abstract

The intra-subject, the inter-operator, and the inter-laboratory variabilities are the main sources of uncertainties in gait analysis, and their effects have been partially described in the literature for adult populations. This study aimed to extend the repeatability and reproducibility analysis to a pediatric population, accounting for the effects induced by the intra-subject variations, the measurement setup, the marker set configuration, and the involved operators in placing markers and EMG electrodes. We evaluated kinematic, kinetic and EMG outputs collected from gait analyses performed on two healthy children in two laboratories, by two operators, and with two marker placement protocols. The two involved centers previously defined a common acquisition procedure based on their routine pipelines. The similarity of kinematic, kinetic, and EMG curves were evaluated by means of the coefficients of the Linear Fit Method, and the Mean Absolute Variability with and without the offset among curves. The inter-operator variability was found to be the main contribution to the overall reproducibility of kinematic and kinetic gait data. On the contrary, the main contribution to the variability of the EMG signals was the intra-subject repeatability that is due to the physiological stride to stride muscle activation variability.

## Introduction

Three-dimensional gait analysis is a common exam performed in motion capture laboratories to quantify movement dysfunctions (Cappozzo et al. 2005), and it may provide information on the development of the neuromuscular system (Hausdorff et al. 1999). Human joints’ kinematics and kinetics are extracted in accordance with clinical protocols (Ferrari et al. 2008) and can be integrated with surface electromyography (EMG) to evaluate muscle recruitment patterns, and neuromuscular control of walking (Granata et al. 2005). The relevant-associated intervals of uncertainties have to be properly evaluated (Schwartz et al. 2004), and depend on: (i) the acquisition systems (Jacobson et al. 1995; Chiari et al. 2005; Windolf et al. 2008); (ii) the calibration volume where gait analyses are performed (Vander Linden et al. 1992; Benedetti et al. 2013; Di Marco et al. 2015, 2016a); (iii) the chosen model and the marker set (Ferrari et al. 2008); (iv) the skills of the operator in placing markers (Gorton et al. 2009; Leigh et al. 2014) and electrodes (Veiersted 1991); (v) the physiological intra-subject stride to stride variability (Meldrum et al. 2014); and, finally, (vi) the soft tissue artefacts (Leardini et al. 2005). Whereas the last two causes of uncertainty are specific of the subject and cannot be eliminated, the first four sources of uncertainties are related to the chosen measurement setup and procedures and should be properly investigated.

Gorton *et al*. (Gorton et al. 2009) evaluated the variability in kinematic data among 12 laboratories and 24 operators studying numerous trials conducted on one healthy adult. They found that variability in marker placement among examiners was the largest quantifiable source of uncertainty when considering gait performed in different centers. Furthermore, they concluded that by introducing a standardized protocol the variability could decrease by 20%. Benedetti et al. (2013) analyzed the variability of kinematic and kinetic data on one healthy adult among seven laboratories. They stated that differences in the collected outcomes were related to the measurement system, software, and the acquisition protocol. Ferrari et al. (2008) compared five widespread marker placement protocols using data collected from two healthy adult subjects and one patient with a knee prosthesis. The authors found a good intra- and inter-protocol repeatability, despite the differences between models and marker sets. The inter-laboratory variability of EMG, only evaluated by (Kleissen et al. 1997), can be reduced using standard instruments, protocols, and processing techniques. It is worth noticing that none of the above-cited studies considered the simultaneous effects of the inter-laboratory and inter-operator variability on the kinematics, kinetics and EMG data. Moreover, to the authors’ knowledge, the inter-operator variability of EMG data during gait has not yet been assessed, and no studies have been performed to evaluate the inter-laboratory and the inter-operator variability on the pediatric population. Considering the differences in gait pattern between adults and children (Hausdorff et al. 1999), and the widespread use of gait analysis in the pediatric population, this study aims to quantify the mentioned sources of uncertainty, in order to assess if the main contribution to the variability of pediatric gait data is related to the variability of the subject, the laboratory, or the operator according to two different biomechanical models. In this perspective, we assess the intra-subject repeatability, the inter-operator, and the inter-laboratory reproducibility of gait variables acquired in healthy children.

## Materials and methods

### Ethic statement

Ethical approval was granted by the Katholieke Universiteit Leuven (KUL) Belgium, and Children’s Hospital ‘Bambino Gesù’ (OPBG) Italy for a sample of two children. Prior to the data collection, the parents read and signed a consent form.

### Experimental procedure

Two typically developing children were enrolled (both male, one of 11 years old, 41.6 kg, and 1.53 m and, one of 8 years old, 36.0 kg and 1.43 m height), with no neurological or orthopedic impairments, history of learning disabilities, and visual impairment.

Gait analyses were performed at the Clinical Motion Analysis Laboratory of the University Hospital Pellenberg at KUL, and at the Movement Analysis and Robotic Laboratory at OPBG. The two subjects visited both labs. In both laboratories, gait analyses were carried out on a 10 m walkway. Subjects were asked to walk barefoot at a self-selected speed. Two experienced therapists per center placed the markers on each subject following the decided protocol, fusing a subset of the Plug-In-Gait (PiG) (Kadaba et al. 1990; Davis et al. 1991) and the Human Body Model (HBM) (Van Den Bogert et al. 2013) at both labs. The PiG protocol consists of 16 retro-reflective markers: posterior and anterior iliac spines (4 markers), lateral femoral epicondyles (2 markers), thighs (2 markers), shanks (2 markers), lateral malleoli (2 markers), second metatarsal heads (2 markers), and calcanei (2 markers). HBM consists of 20 retro-reflective markers: same 16 as PiG except for the second metatarsal heads, complemented with greater trochanter, fifth metatarsal head and second proximal metatarsus. Data were collected using 15 cameras MX (Vicon Motion Systems, Oxford – UK) sampling at 100 Hz at KUL and 8 cameras MX sampling at 200 Hz at OPBG. Synchronized kinetic data were captured using two in-ground AMTI OR-6 force platforms (Advanced Mechanical Technology, Watertown NY – USA), with a full scale of the vertical forces equal to 4.45 kN, at KUL and OPBG. Marker position reconstruction and gait kinematics and kinetics computation were performed at the two labs using Vicon Nexus 1.8.5 (Vicon Motion Systems, Oxford – UK) for the PiG, and D-Flow software (Motekforce Link BV, Amsterdam – The Netherlands) for the HBM.

Electromyography data were recorded using 16 bipolar surface electrodes obtained from eight muscle groups of both side simultaneously of the lower limbs, following the SENIAM guidelines (Stegeman and Hermens 2007): Rectus Femoris, Medial Hamstrings, Anterior Tibialis, Gastrocnemius, Vastus Lateralis, Biceps Femoris, Soleus and Gluteus Medius. EMG signals were collected with a Zero Wire system (Cometa, Milan – Italy, sampling frequency of 1 kHz) at both laboratories.

**Figure 1.**
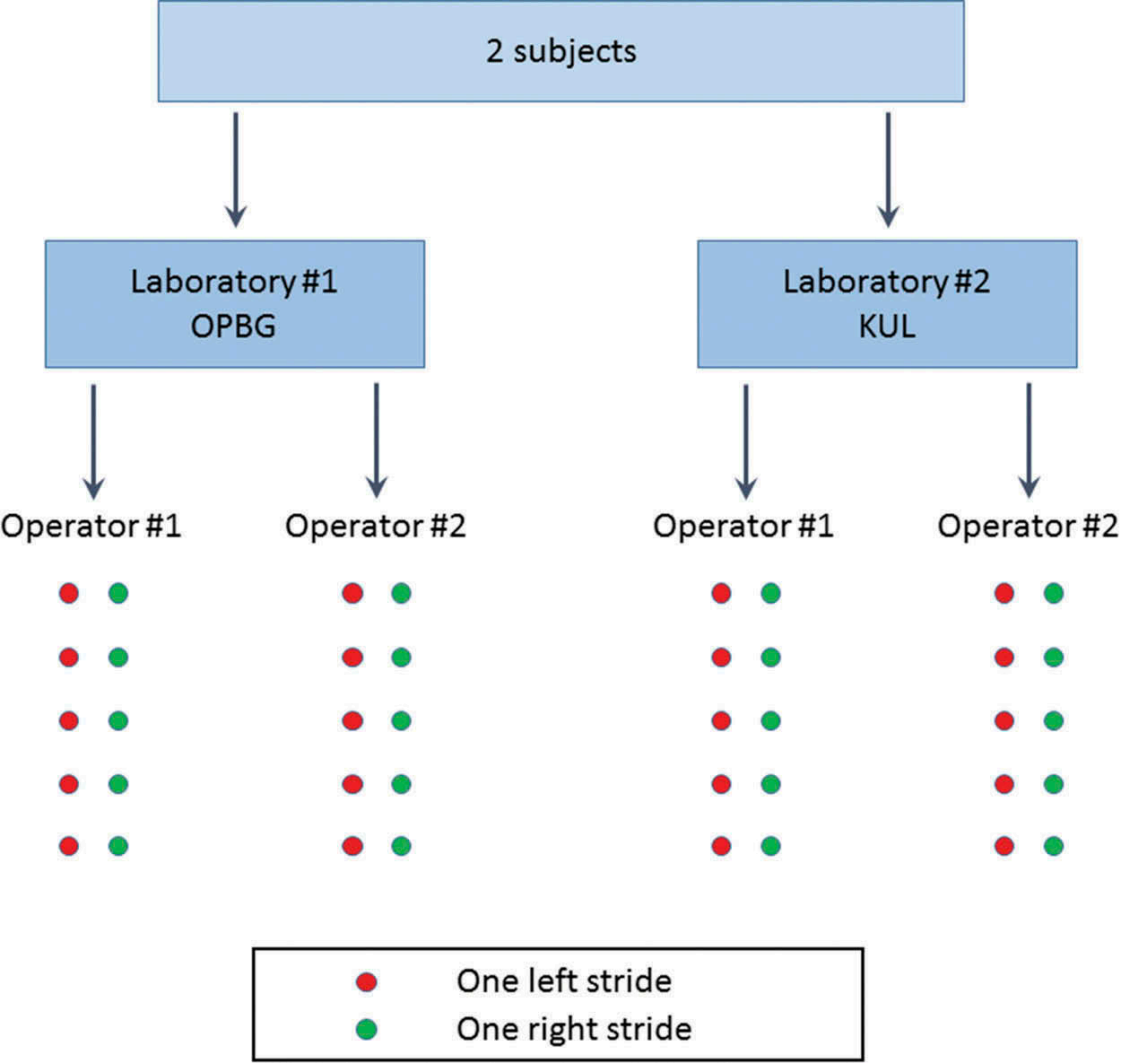
Summary of the data collection procedure. Subjects were equipped with the marker-set obtained merging the Plug-in-Gait and Human Body Model. Kinematics, kinetics, and EMG data were retrieved from each right and left stride.

Five left and five right strides were segmented for each trial and a total of 80 strides were analyzed for each of the two examined biomechanical models (2 subjects x 10 strides x 2 operators x 2 labs). The acquisition process is summarized in Figure 1.

### Data analysis

Raw data for both lower limbs were analyzed for each subject, operator, and laboratory, according to the two protocols. Three-dimensional marker time-histories were smoothed with a Woltring filter – size 30 (Woltring 1985). The kinematic angles were computed for the pelvis segment, the hip, knee and ankle joints in the three anatomical planes; whereas only the sagittal moments of the hip, knee and ankle were considered as relevant kinetic variables. Data were then exported to Matlab (The MathWorks Inc., Natick MA – USA). The raw EMG signals were filtered with a fifth order high-pass Butterworth filter with 20 Hz cut-off frequency and then rectified and smoothed with a 20 ms moving window average (Hershler and Milner 1978). Kinematic, kinetic and EMG outputs were segmented into gait cycles, resampled and normalized at 100 samples per stride.

Firstly, we evaluated the intra-subject repeatability in order to quantify the effect of the stride to stride variability on all gait variables. Then, to quantify the effects induced by different operators, we performed the inter-operator comparison at each laboratory. Finally, we calculated the inter-laboratory reproducibility to assess the effects induced by the measurement setup. These analyses were performed on the kinematic, kinetic and EMG data obtained from both the subjects.

The Linear Fit Method (LFM) (Iosa et al. 2014) and the Mean Absolute Variability (MAV) (Ferrari et al. 2010) were adopted to evaluate the similarity among kinematic, kinetic and EMG waveforms. LFM calculates the linear regression between the dataset under investigation and a reference curve, returning separate information about the scaling factor (a_1_), the weighted averaged offset (a_0_), and the trueness of the linear relation between them (R^2^). When the curve under analysis is equal to the reference curve, the values of LFM parameters are a_1_ = 1, a_0_ = 0 and R^2^ = 1. The determination coefficient R^2^ validates the linear relationship between the curves, and when R^2^ >0.5 the assumption of linearity is considered as valid, with a_1_ and a_0_ considered as meaningful (Iosa et al. 2014). The reference curve changed depending on the analysis: i.e. (i) the mean curve among the five strides for each data-set collected by each operator in each laboratory for the intra-subject repeatability; (ii) the mean curve obtained from five strides and two operators to quantify the inter-operator reproducibility; and (iii) the mean curve obtained from five strides, two operators, and two laboratories to evaluate the inter-laboratory reproducibility. Each single-stride curve within the dataset under investigation was compared with the specific reference curve; and the a_1_, a_0_ and R^2^ were obtained for each comparison. All values were calculated for each of the two subjects separately, and then averaged over subjects. The coefficients a_1_ and a_0_ tend to their ideal values (i.e. 1 and 0, respectively) when comparing n curves with their averaged pattern, as reported in (Iosa et al. 2014). Thus, to have a measure of the variations, it is worthy to report and observe mainly the standard deviations for both a_1_ and a_0,_ rather than their mean values.

As index of data dispersion of the kinematic and kinetic variables, we selected the Mean Absolute Variability (MAV) (Ferrari et al. 2010), which measures the sample-by-sample difference between the maximum and the minimum among the comparing curves. In order to separate the effect induced by the offset from the stride to stride variability, we also computed the MAV removing the offset from the curves and the index was addressed as *offset-corrected* MAV (MAV*_OC_*).

## Results

**Figure 2.**
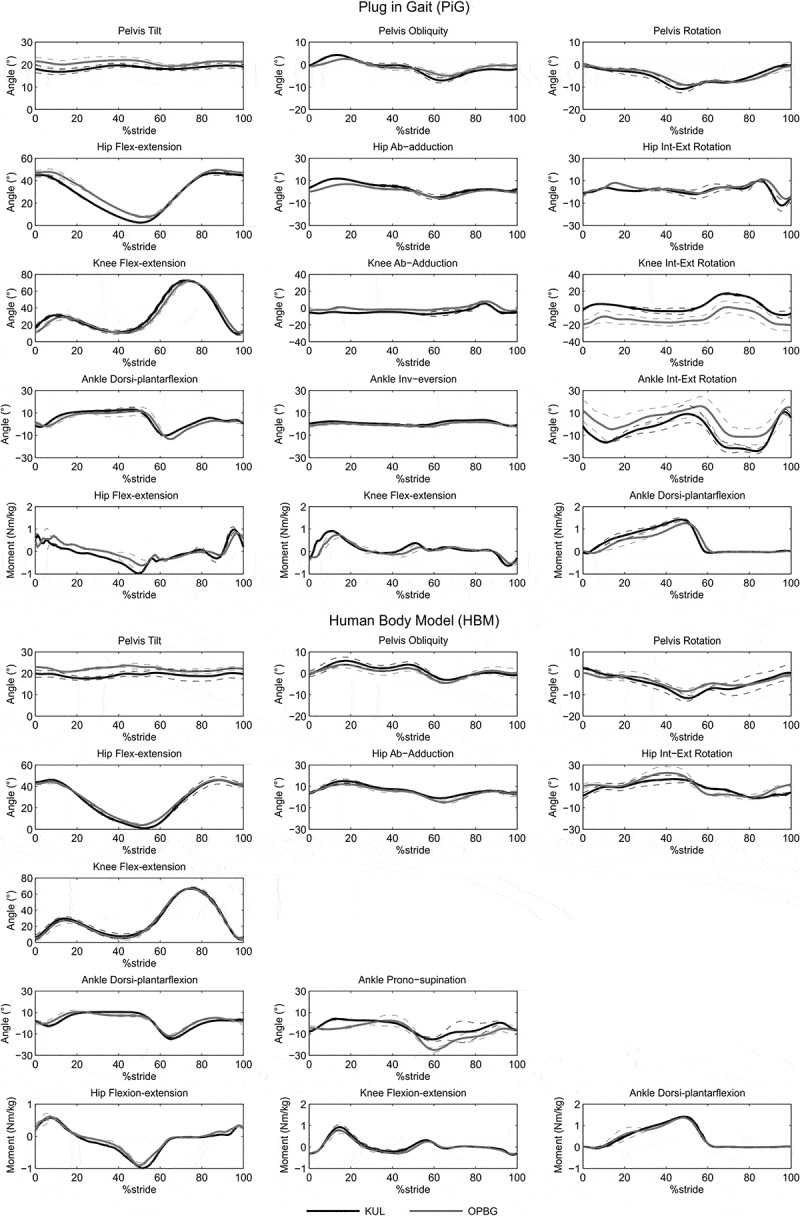
Inter-laboratory reproducibility for subject #1 relative at the two models PiG and HBM: means and standard deviations for kinematic and kinetic variables evaluated at the two labs (KUL, OPBG) for the right lower limb obtained averaging data relative to the two operators per laboratory.

**Figure 3.**
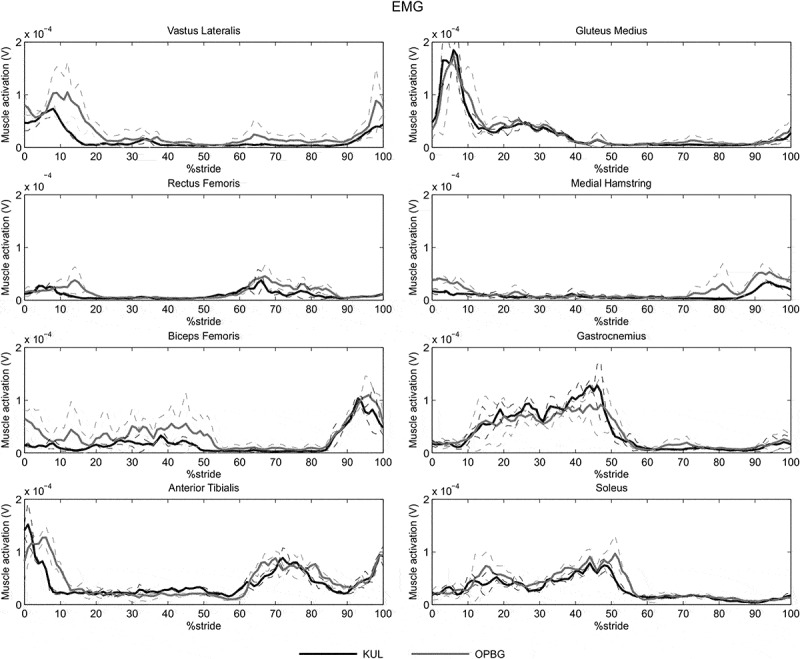
Inter-laboratory reproducibility for subject #1: means and standard deviations for EMG signals at the two labs (KUL, OPBG) for the 8 muscles targeted for the right lower limb obtained averaging data relative to the two operators per laboratory.

Figure 2 shows the kinematic and kinetic time-histories evaluated in the two laboratories on subject #1, which was assumed as representative of both subjects. Figure 3 shows the EMG time-histories of the eight examined muscles on the right lower limb of subject #1.

### Kinematics

Table 1 reports the mean, and the standard deviation of LFM coefficients, MAV and MAV_OC_ calculated for the intra-subject repeatability among the strides, the operators, the laboratories, averaged over both subjects for the kinematics and kinetics evaluated according to both PiG and HBM.

**Table 1.** Intra-subject repeatability: mean and standard deviation values of Linear Fitting Method (LFM) coefficients, Mean Absolute Variability (MAV) and offset-corrected Mean Absolute Variability (MAV_OC_) for the kinematic and kinetic variables evaluated according to the Plug-in-Gait (PiG) and Human Body Model (HBM). [*]: a_0_, MAV and MAV_OC_ are expressed in ° for the kinematics and in Nm/kg for the kinetics.

		PiG						HBM					
			LFM						LFM				
		ROM [*]	a_1_	a_0_ [*]	R^2^	MAV [*]	MAV_OC_ [*]	ROM [*]	a_1_	a_0_ [*]	R^2^	MAV [*]	MAV_OC_ [*]
Right	**Pelvis tilt**	4.1 (1.6)	1.00 (0.72)	0.0 (11.6)	0.55 (0.25)	3.8 (1.8)	2.6 (0.8)	3.9 (1.1)	1.00 (0.44)	0.0 (8.0)	0.65 (0.24)	2.2 (0.9)	1.5 (0.3)
	**Pelvis obl**	9.5 (2.1)	1.00 (0.14)	0.0 (0.7)	0.95 (0.02)	2.6 (0.8)	1.8 (0.6)	7.9 (1.5)	1.00 (0.16)	0.0 (0.7)	0.95 (0.05)	1.9 (0.8)	1.4 (0.3)
	**Pelvis rot**	17.6 (7.5)	1.00 (0.34)	0.0 (2.2)	0.89 (0.10)	6.7 (2.2)	5.2 (1.7)	18.7 (7.1)	1.00 (0.21)	0.0 (1.7)	0.91 (0.06)	4.2 (1.5)	3.0 (0.9)
	**Hip flex/ext**	44.5 (3.5)	1.00 (0.06)	0.0 (1.8)	0.99 (0.00)	5.6 (1.7)	4.5 (1.1)	46.1 (4.3)	1.00 (0.08)	0.0 (1.8)	0.99 (0.01)	3.6 (1.3)	3.1 (1.0)
	**Hip ab/add**	14.4 (3.0)	1.00 (0.20)	0.0 (1.1)	0.93 (0.03)	4.1 (0.8)	3.4 (0.9)	16.1 (2.1)	1.00 (0.14)	0.0 (1.2)	0.96 (0.03)	2.9 (1.3)	2.3 (0.7)
	**Hip rot**	23.6 (9.8)	1.00 (0.12)	0.0 (1.2)	0.82 (0.10)	6.1 (1.5)	5.5 (1.4)	25.1 (6.5)	1.00 (0.15)	0.0 (1.8)	0.95 (0.03)	4.5 (2.1)	3.5 (1.2)
	**Knee flex/ext**	62.1 (3.3)	1.00 (0.05)	0.0 (1.8)	0.99 (0.01)	7.3 (3.0)	6.8 (2.7)	62.8 (3.9)	1.00 (0.06)	0.0 (1.9)	0.99 (0.01)	4.3 (2.5)	3.7 (2.3)
	**Knee ab/ad**	16.3 (4.8)	1.00 (0.17)	0.0 (0.9)	0.85 (0.10)	3.7 (1.3)	3.6 (1.2)	-	-	-	-	-	-
	**Knee rot**	24.7 (4.7)	1.00 (0.12)	0.0 (1.4)	0.92 (0.04)	4.9 (1.6)	4.2 (1.2)	-	-	-	-	-	-
	**Ankle dor/pl**	25.5 (3.2)	1.00 (0.11)	0.0 (1.1)	0.95 (0.01)	4.9 (1.6)	4.3 (1.5)	24.8 (4.1)	1.00 (0.14)	0.0 (1.4)	0.97 (0.02)	3.3 (1.1)	2.7 (0.6)
	**Ankle inv/ev**	5.7 (1.5)	1.00 (0.11)	0.0 (0.3)	0.95 (0.01)	1.1 (0.4)	1.0 (0.3)	24.9(4.1)	1.00 (0.13)	0.0 (1.3)	0.93 (0.05)	4.5 (1.7)	3.9 (1.4)
	**Ankle rot**	34.0 (4.4)	1.00 (0.10)	0.0 (1.6)	0.96 (0.01)	6.9 (2.0)	6.0 (1.3)	-	-	-	-	-	-
	**Hip moment**	1.87 (0.30)	1.00 (0.10)	0.0 (0.0)	0.94 (0.02)	0.30 (0.07)	0.29 (0.07)	1.36 (0.26)	1.00 (0.10)	0.0 (0.0)	0.98 (0.01)	0.12 (0.04)	0.12 (0.04)
	**Knee moment**	1.36 (0.21)	1.00 (0.09)	0.0 (0.0)	0.96 (0.02)	0.19 (0.03)	0.19 (0.04)	1.16 (0.17)	1.00 (0.11)	0.0 (0.0)	0.97 (0.01)	0.10 (0.04)	0.10 (0.04)
	**Ankle moment**	1.32 (0.18)	1.00 (0.05)	0.0 (0.0)	0.99 (0.01)	0.15 (0.05)	0.16 (0.04)	1.30 (0.19)	1.10 (0.03)	0.0 (0.0)	0.98 (0.01)	0.08 (0.04)	0.09 (0.04)
Left	**Hip flex/ext**	44.2 (4.4)	1.00 (0.06)	0.0 (1.7)	0.99 (0.01)	5.8 (2.1)	4.8 (1.8)	46.7 (4.6)	1.00 (0.05)	0.0 (2.4)	0.99 (0.01)	4.8 (2.6)	3.5 (1.8)
	**Hip ab/add**	12.1 (2.2)	1.00 (0.18)	0.0 (1.0)	0.97 (0.12)	3.5 (0.9)	3.3 (0.7)	14.9 (2.1)	1.00 (0.15)	0.0 (1.0)	0.91 (0.06)	3.1 (2.2)	2.6 (1.1)
	**Hip rot**	21.6 (3.7)	1.00 (0.11)	0.0 (1.2)	0.86 (0.08)	5.4 (0.9)	5.0 (0.8)	23.0 (5.9)	1.00 (0.17)	0.0 (1.5)	0.92 (0.04)	5.2 (2.2)	4.4 (2.3)
	**Knee flex/ext**	62.2 (3.8)	1.00 (0.05)	0.0 (2.3)	0.99 (0.02)	7.9 (2.7)	7.1 (2.1)	64.3 (4.4)	1.00 (0.02)	0.0 (1.9)	0.99 (0.00)	5.3 (2.9)	4.4 (2.8)
	**Knee ab/ad**	14.3 (4.2)	1.00 (0.24)	0.0 (0.7)	0.89 (0.12)	3.3 (1.2)	3.4 (1.1)	-	-	-	-	-	-
	**Knee rot**	22.8 (4.3)	1.00 (0.11)	0.0 (1.1)	0.97 (0.03)	4.6 (1.1)	3.8 (1.1)	-	-	-	-	-	-
	**Ankle dor/pl**	21.2 (4.1)	1.00 (0.13)	0.0 (1.4)	0.92 (0.04)	4.7 (0.7)	4.0 (0.9)	22.8 (4.9)	1.00 (0.09)	0.0 (0.7)	0.97 (0.01)	2.5 (1.2)	2.2 (1.0)
	**Ankle inv/ev**	4.6 (1.7)	1.00 (0.09)	0.0 (0.3)	0.93 (0.04)	1.2 (0.6)	0.9 (0.3)	23.7 (4.5)	1.00 (0.16)	0.0 (2.2)	0.91 (0.05)	5.6 (3.1)	4.1 (1.7)
	**Ankle rot**	31.9 (5.2)	1.00 (0.09)	0.0 (2.2)	0.93 (0.04)	7.7 (2.5)	6.3 (1.0)	-	-	-	-	-	-
	**Hip moment**	1.86 (0.36)	1.00 (0.12)	0.0 (0.0)	0.91 (0.08)	0.31 (0.14)	0.33 (0.16)	1.36 (0.23)	1.00 (0.08)	0.0 (0.0)	0.98 (0.11)	0.11 (0.02)	0.11 (0.02)
	**Knee moment**	1.17 (0.22)	1.00 (0.14)	0.0 (0.0)	0.93 (0.06)	0.19 (0.03)	0.20 (0.03)	1.14 (0.20)	1.00 (0.18)	0.0 (0.0)	0.96 (0.03)	0.13 (0.04)	0.13 (0.05)
	**Ankle moment**	1.37 (0.26)	1.00 (0.05)	0.0 (0.0)	0.99 (0.02)	0.19 (0.13)	0.22 (0.13)	1.38 (0.16)	1.00 (0.03)	0.0 (0.0)	0.98 (0.01)	0.10 (0.04)	0.10 (0.05)

For the PiG model, the intra-subject analysis in the sagittal plane yielded the highest averaged R^2^ for hip and knee flexion/extension (R^2^ = 0.99); the ankle dorsiflexion showed lower values of the determination coefficient (R^2^ = 0.92–0.95) than the hip and knee flexion/extension. The pelvic tilt showed the lowest mean value of the intra-subject repeatability (R^2^ = 0.55). Regarding the coronal and transverse planes, the R^2^ ranged between 0.82 and 0.97. The standard deviation of the scaling factor (a_1_) was lower than 0.34 for all the examined kinematic variables with the exception of pelvic tilt that showed a standard deviation of a_1_ equal to 0.72. The average MAV was 5°, while the maximum difference between MAV, and the offset-corrected MAV_OC_ was less than 1.5°, indicating that most of the intra-subject variability was due to variation in the shape of the curve, rather than offset.

The intra-subject repeatability was in general better for HBM than for PiG. In fact, the mean R^2^ related to the HBM in the sagittal plane was 0.99 for the hip, and the knee angles, while was 0.97 for the ankle angles. The lowest values of R^2^ was found for the pelvic tilt (R^2^ = 0.65). Regarding the coronal and transverse planes, the R^2^ ranged between 0.91 and 0.98. The standard deviation of the scaling factor (a_1_) was lower than 0.21 for all the examined kinematic variables, with the exception of pelvic tilt that showed a standard deviation of a_1_ equal to 0.44. The average MAV was approximately 3.5°, and the maximum difference between MAV and MAV_OC_ was 1.2°.

The inter-operator LFM coefficients, MAV and MAV_OC_ averaged over both subjects and evaluated according to the two models, are reported in Tables 2 and 3. The results relative to the inter-operator comparison showed lower reproducibility with respect to the intra-subject ones. For the PiG model, sagittal hip, knee and ankle kinematics of both subjects presented R^2^ values from 0.90 to 0.99 (Table 2). The standard deviation of the scaling factor a_1_ was always lower than 0.15. MAV values ranged between 1.4° and 4.4°, and the difference between MAV and MAV_OC_ was always lower than about 1°. An exception was found for the right hip kinematics at KUL: MAV and MAV_OC_ were 3.7° and 1.0°, respectively. The R^2^ of pelvic tilt was 0.47 and 0.49 for KUL and OPBG labs, respectively. MAV and MAV_OC_ obtained for pelvic tilt were 2.7° and 0.8° at KUL and 1.3° and 0.4° at OPBG. For the frontal and transverse planes, the R^2^ of kinematic variables ranged from 0.75 to 0.95 for both labs. The standard deviation of the scaling factor was, in the worst case, equal to 0.38, which was obtained for the knee abduction (Table 2, KUL). At OPBG, the knee and ankle rotations of both subjects were the most affected by the offset. In particular, MAV ranged from 1.6° to 8.5°, and MAV_OC_ from 1.3° to 1.9° for knee rotation, whereas for ankle rotation MAV was in the range between 4.7° and 11.2°, and MAV_OC_ between 1.9° and 3.3°. The results of the inter-operator comparison according to the HBM model were very similar to those of PiG (Table 3). The R^2^ of the sagittal kinematics always ranged between 0.95 and 0.99. The standard deviation of the scaling factor never exceeded 0.15. The highest values of MAV and MAV_OC_ were 3.4° and 0.9°, and obtained for the left hip flexion at KUL (Table 3). In other cases, the difference between MAV and MAV_OC_ was always lower than 1°. As regards the pelvic tilt, the R^2^ were 0.48 and 0.63 at KUL and OPBG, respectively. For the coronal and transverse plane variables, the R^2^ ranged from 0.83 to 0.94, and the averaged scaling factor was always equal to 1 with the highest value of standard deviation equal to 0.31. The highest values of MAV and MAV_OC_ were 6.0° and 2.1° for the hip rotation at KUL.

**Table 2.** Inter-operator reproducibility: mean and standard deviation values of Linear Fitting Method (LFM) coefficients, Mean Absolute Variability (MAV) and offset-corrected Mean Absolute Variability (MAV_OC_) for the kinematic and kinetic variables evaluated at KUL and OPBG, according to the Plug-in-Gait (PiG). [*]: a_0_, MAV and MAV_OC_ are expressed in ° for the kinematics and in Nm/kg for the kinetics.

		PiG											
		KUL						OPBG					
			LFM						LFM				
		ROM [*]	a_1_	a_0_ [*]	R^2^	MAV [*]	MAV_OC_ [*]	ROM [*]	a_1_	a_0_ [*]	R^2^	MAV [*]	MAV_OC_ [*]
Right	**Pelvis tilt**	4.2 (1.7)	1.00 (0.82)	0.0 (14.3)	0.47 (0.26)	2.7	0.8	4.1 (1.5)	1.00 (0.96)	0.0 (11.7)	0.49 (0.31)	1.3	0.4
	**Pelvis obl**	11.0 (1.9)	1.00 (0.19)	0.0 (0.9)	0.94 (0.05)	1.4	0.5	8.1 (1.3)	1.00 (0.15)	0.0 (0.9)	0.92 (0.07)	1.1	0.6
	**Pelvis rot**	17.9 (6.7)	1.00 (0.26)	0.0 (1.9)	0.87 (0.14)	1.2	1.2	17.3 (8.4)	1.00 (0.28)	0.0 (2.5)	0.86 (0.11)	1.6	1.5
	**Hip flex/ext**	44.7 (3.1)	1.00 (0.07)	0.0 (2.3)	0.99 (0.01)	3.7	1.0	44.4 (3.9)	1.00 (0.07)	0.0 (2.4)	0.99 (0.01)	2.0	1.4
	**Hip ab/add**	15.1 (3.1)	1.00 (0.22)	0.0 (1.5)	0.95 (0.05)	2.2	0.8	13.6 (2.8)	1.00 (0.22)	0.0 (1.6)	0.90 (0.09)	1.0	0.9
	**Hip rot**	28.4 (11.8)	1.00 (0.35)	0.0 (2.0)	0.77 (0.15)	5.1	4.1	18.8 (2.9)	1.00 (0.16)	0.0 (1.8)	0.78 (0.13)	2.8	1.1
	**Knee flex/ext**	61.4 (3.7)	1.00 (0.07)	0.0 (2.8)	0.98 (0.03)	2.5	1.6	62.9 (2.7)	1.00 (0.04)	0.0 (1.6)	0.98 (0.02)	2.0	1.5
	**Knee ab/ad**	18.3 (5.7)	1.00 (0.38)	0.0 (2.1)	0.76 (0.25)	3.1	3.1	14.4 (2.8)	1.00 (0.21)	0.0 (0.7)	0.82 (0.14)	1.4	1.1
	**Knee rot**	23.5 (4.8)	1.00 (0.19)	0.0 (2.1)	0.90 (0.08)	2.5	2.0	26.0 (4.4)	1.00 (0.14)	0.0 (5.4)	0.92 (0.05)	8.5	1.9
	**Ankle dor/pl**	24.2 (3.0)	1.00 (0.13)	0.0 (1.8)	0.94 (0.05)	1.5	0.9	26.7 (2.9)	1.00 (0.13)	0.0 (0.9)	0.92 (0.07)	2.2	2.0
	**Ankle inv/ev**	6.0 (1.6)	1.00 (0.21)	0.0 (0.6)	0.94 (0.03)	0.6	0.6	5.3 (1.4)	1.00 (0.14)	0.0 (0.8)	0.93 (0.05)	1.4	0.4
	**Ankle rot**	35.4 (3.8)	1.00 (0.15)	0.0 (3.8)	0.95 (0.02)	4.6	2.8	32.6 (4.5)	1.00 (0.09)	0.0 (6.1)	0.93 (0.05)	11.2	1.9
	**Hip moment**	1.89 (0.27)	1.00 (0.07)	0.0 (0.0)	0.92 (0.03)	0.08	0.08	1.85 (0.34)	1.00 (0.20)	0.0 (0.0)	0.81 (0.15)	0.11	0.11
	**Knee moment**	1.50 (0.12)	1.00 (0.18)	0.0 (0.0)	0.94 (0.05)	0.04	0.04	1.23 (0.20)	1.00 (0.12)	0.0 (0.0)	0.88 (0.09)	0.07	0.07
	**Ankle moment**	1.41 (0.13)	1.00 (0.04)	0.0 (0.0)	0.98 (0.02)	0.06	0.06	1.23 (0.18)	1.00 (0.10)	0.0 (0.2)	0.95 (0.04)	0.07	0.07
Left	**Hip flex/ext**	46.4 (4.4)	1.00 (0.07)	0.0 (2.3)	0.99 (0.02)	4.0	1.4	41.9 (3.9)	1.00 (0.08)	0.0 (2.8)	0.98 (0.02)	2.8	1.8
	**Hip ab/add**	12.1 (2.2)	1.00 (0.19)	0.0 (1.0)	0.91 (0.07)	1.9	1.1	11.2 (2.3)	1.00 (0.24)	0.0 (1.7)	0.86 (0.08)	2.4	1.1
	**Hip rot**	21.6 (3.7)	1.00 (0.24)	0.0 (2.4)	0.81 (0.12)	3.5	2.3	20.5 (2.3)	1.00 (0.12)	0.0 (1.5)	0.80 (0.12)	2.6	1.9
	**Knee flex/ext**	62.2 (3.8)	1.00 (0.06)	0.0 (2.5)	0.97 (0.02)	1.9	1.8	61.8 (3.9)	1.00 (0.07)	0.0 (3.1)	0.97 (0.04)	4.4	3.4
	**Knee ab/ad**	14.3 (4.2)	1.00 (0.35)	0.0 (1.5)	0.75 (0.14)	2.5	2.0	12.7 (2.8)	1.00 (0.24)	0.0 (1.2)	0.78 (0.17)	2.0	0.7
	**Knee rot**	22.8 (4.3)	1.00 (0.10)	0.0 (1.9)	0.91 (0.03)	3.4	1.3	21.7 (3.7)	1.00 (0.15)	0.0 (1.5)	0.91 (0.06)	1.6	1.3
	**Ankle dor/pl**	21.2 (4.1)	1.00 (0.15)	0.0 (1.6)	0.90 (0.05)	1.4	1.4	20.8 (4.7)	1.00 (0.15)	0.0 (1.6)	0.90 (0.10)	2.0	1.8
	**Ankle inv/ev**	4.6 (1.7)	1.00 (0.16)	0.0 (0.5)	0.84 (0.06)	0.5	0.4	3.9 (1.7)	1.00 (0.22)	0.0 (0.3)	0.92 (0.06)	0.6	0.5
	**Ankle rot**	31.9 (5.2)	1.00 (0.11)	0.0 (3.5)	0.85 (0.06)	2.7	2.0	34.8 (4.9)	1.00 (0.21)	0.0 (3.0)	0.92 (0.06)	4.7	3.3
	**Hip moment**	1.86 (0.36)	1.00 (0.07)	0.0 (0.0)	0.92 (0.07)	0.07	0.07	1.79 (0.29)	1.00 (0.18)	0.0 (0.1)	0.82 (0.22)	0.12	0.12
	**Knee moment**	1.17 (0.22)	1.00 (0.14)	0.0 (0.0)	0.94 (0.04)	0.06	0.05	1.08 (0.21)	1.00 (0.18)	0.0 (0.0)	0.86 (0.12)	0.07	0.07
	**Ankle moment**	1.37 (0.26)	1.00 (0.05)	0.0 (0.0)	0.96 (0.02)	0.06	0.06	1.30 (0.34)	1.00 (0.23)	0.0 (0.0)	0.93 (0.10)	0.08	0.07

**Table 3.** Inter-operator reproducibility: mean and standard deviation values of Linear Fitting Method (LFM) coefficients, Mean Absolute Variability (MAV) and offset-corrected Mean Absolute Variability (MAV_OC_) for the kinematic and kinetic variables evaluated at KUL and OPBG, according to the Human Body Model (HBM). [*]: a_0_, MAV and MAV_OC_ are expressed in ° for the kinematics and in Nm/kg for the kinetics.

		HBM											
		KUL						OPBG					
			LFM						LFM				
		ROM [*]	a_1_	a_0_ [*]	R^2^	MAV [*]	MAV_OC_ [*]	ROM [*]	a_1_	a_0_ [*]	R^2^	MAV [*]	MAV_OC_ [*]
Right	**Pelvis tilt**	3.4 (1.0)	1.00 (0.30)	0.0 (5.0)	0.48 (0.21)	3.4	0.8	4.5 (1.0)	1.00 (0.33)	0.0 (5.8)	0.63 (0.23)	1.3	0.7
	**Pelvis obl**	8.2 (81.4)	1.00 (0.18)	0.0 (1.0)	0.92 (0.06)	1.9	0.8	7.6 (1.5)	1.00 (0.22)	0.0 (1.1)	0.86 (0.09)	1.3	0.7
	**Pelvis rot**	19.7 (6.2)	1.00 (0.23)	0.0 (1.5)	0.89 (0.08)	2.5	2.6	17.6 (8.1)	1.00 (0.19)	0.0 (1.9)	0.83 (0.11)	1.6	1.3
	**Hip flex/ext**	47.0 (4.2)	1.00 (0.08)	0.0 (2.1)	0.99 (0.01)	2.8	1.7	45.1 (4.2)	1.00 (0.08)	0.0 (2.4)	0.99 (0.01)	2.0	1.7
	**Hip ab/add**	15.8 (1.7)	1.00 (0.17)	0.0 (1.3)	0.94 (0.06)	1.6	1.0	16.5 (2.6)	1.00 (0.19)	0.0 (1.4)	0.94 (0.06)	1.4	1.1
	**Hip rot**	23.7 (6.5)	1.00 (0.14)	0.0 (3.4)	0.94 (0.04)	6.0	2.1	26.7 (6.3)	1.00 (0.24)	0.0 (1.9)	0.93 (0.09)	3.3	3.1
	**Knee flex/ext**	61.2 (4.2)	1.00 (0.06)	0.0 (2.8)	0.99 (0.02)	2.5	2.0	64.8 (2.4)	1.00 (0.05)	0.0 (2.3)	0.99 (0.00)	2.8	2.7
	**Knee ab/ad**	-	-	-	-	-	-		-	-	-	-	-
	**Knee rot**	-	-	-	-	-	-		-	-	-	-	-
	**Ankle dor/pl**	25.3 (3.8)	1.00 (0.14)	0.0 (1.7)	0.97 (0.03)	1.0	0.7	24.1 (4.4)	1.00 (0.15)	0.0 (1.3)	0.95 (0.03)	1.5	1.4
	**Ankle inv/ev**	22.6 (3.9)	1.00 (0.23)	0.0 (1.2)	0.85 (0.16)	2.9	2.9	27.6 (4.6)	1.00 (0.15)	0.0 (2.6)	0.92 (0.06)	3.3	2.2
	**Ankle rot**	-	-	-	-	-	-		-	-	-	-	-
	**Hip moment**	1.38 (0.27)	1.00 (0.11)	0.0 (0.0)	0.98 (0.01)	0.04	0.04	1.32 (0.26)	1.00 (0.11)	0.0 (0.0)	0.97 (0.03)	0.05	0.05
	**Knee moment**	1.23 (0.15)	1.00 (0.11)	0.0 (0.0)	0.97 (0.02)	0.05	0.04	1.08 (0.16)	1.00 (0.15)	0.0 (0.0)	0.97 (0.02)	0.06	0.06
	**Ankle moment**	1.35 (0.17)	1.00 (0.04)	0.0 (0.0)	0.98 (0.02)	0.04	0.04	1.23 (0.20)	1.00 (0.03)	0.0 (0.0)	0.97 (0.03)	0.06	0.06
Left	**Hip flex/ext**	47.0 (4.6)	1.00 (0.07)	0.0 (2.3)	0.99 (0.01)	3.4	0.9	46.3 (5.1)	1.00 (0.07)	0.0 (3.2)	0.99 (0.01)	2.6	1.9
	**Hip ab/add**	15.8 (1.7)	1.00 (0.24)	0.0 (1.2)	0.89 (0.12)	2.2	1.6	13.9 (2.2)	1.00 (0.18)	0.0 (1.6)	0.84 (0.10)	2.1	1.4
	**Hip rot**	23.7 (6.5)	1.00 (0.31)	0.0 (2.9)	0.91 (0.09)	4.3	3.4	22.3 (5.3)	1.00 (0.17)	0.0 (1.5)	0.92 (0.06)	2.0	1.3
	**Knee flex/ext**	61.8 (3.8)	1.00 (0.03)	0.0 (1.1)	0.99 (0.01)	1.8	1.6	67.2 (3.3)	1.00 (0.06)	0.0 (3.3)	0.97 (0.02)	4.5	3.9
	**Knee ab/ad**	-	-	-	-	-	-	-	-	-	-	-	-
	**Knee rot**	-	-	-	-	-	-	-	-	-	-	-	-
	**Ankle dor/pl**	24.8 (5.6)	1.00 (0.15)	0.0 (1.1)	0.95 (0.02)	1.5	1.3	20.4 (2.4)	1.00 (0.07)	0.0 (1.5)	0.95 (0.02)	2.6	1.1
	**Ankle inv/ev**	24.4 (4.8)	1.00 (0.26)	0.0 (2.6)	0.84 (0.14)	4.4	3.7	22.8 (4.2)	1.00 (0.19)	0.0 (2.7)	0.90 (0.06)	3.6	2.2
	**Ankle rot**	-	-	-	-	-	-	-	-	-	-	-	-
	**Hip moment**	1.42 (0.20)	1.00 (0.08)	0.0 (0.0)	0.98 (0.01)	0.05	0.05	1.29 (0.26)	1.00 (0.08)	0.0 (0.0)	0.96 (0.04)	0.06	0.06
	**Knee moment**	1.17 (0.22)	1.00 (0.20)	0.0 (0.0)	0.95 (0.04)	0.05	0.05	1.11 (0.18)	1.00 (0.15)	0.0 (0.0)	0.94 (0.04)	0.08	0.07
	**Ankle moment**	1.41 (0.15)	1.00 (0.05)	0.0 (0.0)	0.98 (0.02)	0.05	0.04	1.33 (0.16)	1.00 (0.04)	0.0 (0.0)	0.98 (0.02)	0.05	0.04

For the inter-laboratory comparison, the LFM coefficients, MAVs and MAV_OC_ averaged over both subjects and evaluated according to both models are reported in Table 4. R^2^ values were, in general, lower than those obtained for both the intra-subject and inter-operator analysis: for the kinematics evaluated with the PiG model, the R^2^ of the joint angles in the sagittal plane ranged between 0.89 and 0.98. The standard deviation of the scaling factor was always lower than 0.17. The maximum values of MAV (9.0°) and MAV_OC_ (2.2°) were found for hip flexion. The inter-laboratory R^2^ of pelvic tilt was 0.43, and MAV and MAV_OC_ were 6.5° and 0.5°, respectively. The R^2^ ranged between 0.72 and 0.90 for the kinematics in frontal and transverse planes. Left knee abduction showed the highest standard deviation of the scaling factor (0.40). As for the inter-operator comparison, the knee and ankle rotation showed the highest values of MAV and MAV_OC_. In particular, MAV ranged from 9.9° to 17.2°, and MAV_OC_ from 2.0° to 2.4° for knee rotation, whereas MAV values were in the range between 13.0° and 16.6°, and MAV_OC_ between 2.9° and 3.6° for ankle rotation. Similar as for PiG, the joint angles of hip, knee, and ankle obtained according to HBM (Table 4) showed the highest values of the R^2^ in the sagittal plane (R^2^ ≥ 0.90); the standard deviation of the scaling factor was lower than 0.19. The maximum MAV and MAV_OC_ values were 6.4° and 1.5°, respectively, for right hip flexion/extension. The R^2^ of pelvic tilt was equal to 0.45 and MAV and MAV_OC_ were 5.3° and 0.8°. In the coronal and transverse planes, the R^2^ ranged between 0.83 and 0.93. The maximum standard deviation of the scaling factor was obtained for pelvis rotation (0.28). MAV ranged between 1.1° and 6.6° and MAV_OC_ ranged between 0.8° and 2.4°.

**Table 4.** Inter-laboratory reproducibility: mean and standard deviation values of Linear Fitting Method (LFM) coefficients, Mean Absolute Variability (MAV) and offset-corrected Mean Absolute Variability (MAV_OC_) for the kinematic and kinetic variables evaluated according to the Plug-in-Gait (PiG) and the Human Body Model (HBM). [*]: a_0_, MAV and MAV_OC_ are expressed in ° for the kinematics and in Nm/kg for the kinetics.

		PiG						HBM					
			LFM						LFM				
		ROM [*]	a_1_	a_0_ [*]	R^2^	MAV [*]	MAV_OC_ [*]	ROM [*]	a_1_	a_0_ [*]	R^2^	MAV [*]	MAV_OC_ [*]
Right	**Pelvis tilt**	4.1 (1.6)	0.65 (1.28)	4.9 (20.5)	0.43 (0.24)	6.5	0.5	3.9 (1.1)	1.00 (0.55)	0.0 (9.1)	0.45 (0.25)	5.3	0.8
	**Pelvis obl**	9.5 (2.1)	0.99 (0.25)	0.0 (0.8)	0.90 (0.07)	2.3	1.0	7.9 (1.5)	1.00 (0.23)	0.0 (1.2)	0.84 (0.51)	1.1	0.9
	**Pelvis rot**	17.6 (7.5)	0.99 (0.30)	0.0 (2.6)	0.83 (0.17)	3.8	0.9	18.7 (7.1)	1.00 (0.28)	0.0 (1.9)	0.84 (0.10)	2.1	1.7
	**Hip flex/ext**	44.5 (3.5)	1.00 (0.07)	0.0 (4.4)	0.98 (0.02)	9.0	2.2	46.1 (4.3)	1.00 (0.08)	0.0 (4.5)	0.99 (0.01)	6.4	1.5
	**Hip ab/add**	14.4 (3.0)	1.00 (0.25)	0.0 (2.6)	0.90 (0.08)	6.0	1.1	16.1 (2.1)	1.00 (0.18)	0.0 (1.9)	0.93 (0.06)	2.1	0.8
	**Hip rot**	23.6 (9.8)	1.00 (0.35)	0.0 (2.1)	0.72 (0.17)	7.3	2.8	25.1 (6.5)	1.00 (0.22)	0.0 (3.5)	0.89 (0.10)	4.1	2.4
	**Knee flex/ext**	62.1 (3.3)	1.00 (0.07)	0.0 (3.3)	0.97 (0.04)	7.1	3.6	62.8 (3.9)	1.00 (0.07)	0.0 (3.3)	0.98 (0.01)	2.9	2.6
	**Knee ab/ad**	16.3 (4.8)	1.00 (0.33)	0.0 (2.6)	0.77 (0.19)	7.4	1.9	-	-	-	-	-	-
	**Knee rot**	24.7 (4.7)	1.00 (0.20)	0.0 (7.2)	0.88 (0.09)	17.2	2.0	-	-	-	-	-	-
	**Ankle dor/pl**	25.5 (3.2)	1.00 (0.14)	0.0 (2.5)	0.90 (0.08)	5.9	2.0	24.8 (4.1)	1.00 (0.17)	0.0 (1.5)	0.94 (0.04)	2.0	1.9
	**Ankle inv/ev**	5.7 (1.5)	1.00 (0.22)	0.0 (1.0)	0.90 (0.08)	2.4	0.6	24.9(4.1)	1.00 (0.22)	0.0 (3.2)	0.85 (0.16)	6.6	2.8
	**Ankle rot**	34.0 (4.4)	1.00 (0.17)	0.0 (6.3)	0.90 (0.08)	16.6	3.6	-	-	-	-	-	-
	**Hip moment**	1.87 (0.30)	1.00 (0.16)	0.0 (0.1)	0.83 (0.14)	0.25	0.11	1.36 (0.26)	1.00 (0.12)	0.0 (0.0)	0.97 (0.02)	0.05	0.05
	**Knee moment**	1.36 (0.21)	1.00 (0.16)	0.0 (0.0)	0.86 (0.12)	0.16	0.11	1.16 (0.17)	1.00 (0.14)	0.0 (0.0)	0.96 (0.03)	0.05	0.05
	**Ankle moment**	1.32 (0.18)	1.00 (0.14)	0.0 (0.0)	0.95 (0.05)	0.20	0.15	1.30 (0.19)	1.00 (0.07)	0.0 (0.0)	0.98 (0.03)	0.05	0.05
Left	**Hip flex/ext**	44.2 (4.4)	1.00 (0.10)	0.0 (4.0)	0.98 (0.02)	9.1	1.9	46.7 (4.6)	1.00 (0.08)	0.0 (4.1)	0.99 (0.01)	5.5	2.0
	**Hip ab/add**	12.1 (2.2)	1.00 (0.25)	0.0 (2.0)	0.85 (0.11)	5.0	1.4	14.9 (2.1)	1.00 (0.22)	0.0 (1.4)	0.83 (0.11)	1.6	1.4
	**Hip rot**	21.6 (3.7)	1.00 (0.20)	0.0 (2.0)	0.75 (0.15)	5.2	2.0	23.0 (5.9)	1.00 (0.27)	0.0 (2.3)	0.88 (0.11)	2.1	1.9
	**Knee flex/ext**	62.2 (3.8)	1.00 (0.07)	0.0 (3.3)	0.97 (0.03)	7.0	2.7	64.3 (4.4)	1.00 (0.06)	0.0 (3.2)	0.97 (0.02)	4.8	3.4
	**Knee ab/ad**	14.3 (4.2)	1.00 (0.40)	0.0 (1.7)	0.75 (0.19)	5.3	1.7	-	-	-	-	-	-
	**Knee rot**	22.8 (4.3)	1.00 (0.20)	0.0 (4.2)	0.90 (0.06)	9.9	2.4	-	-	-	-	-	-
	**Ankle dor/pl**	21.2 (4.1)	1.00 (0.17)	0.0 (2.0)	0.89 (0.10)	4.3	1.1	22.8 (4.9)	1.00 (0.19)	0.0 (2.0)	0.90 (0.09)	4.1	3.0
	**Ankle inv/ev**	4.6 (1.7)	1.00 (0.38)	0.0 (1.0)	0.88 (0.08)	2.9	0.7	23.7 (4.5)	1.00 (0.24)	0.0 (3.2)	0.84 (0.12)	3.6	1.8
	**Ankle rot**	31.9 (5.2)	1.00 (0.18)	0.0 (6.2)	0.89 (0.08)	13.0	2.9	-	-	-	-	-	-
	**Hip moment**	1.86 (0.36)	1.00 (0.22)	0.0 (0.1)	0.83 (0.19)	0.25	0.14	1.36 (0.23)	1.00 (0.11)	0.0 (0.0)	0.96 (0.04)	0.07	0.06
	**Knee moment**	1.17 (0.22)	1.00 (0.19)	0.0 (0.0)	0.86 (0.12)	0.16	0.09	1.14 (0.20)	1.00 (0.19)	0.0 (0.1)	0.91 (0.07)	0.10	0.09
	**Ankle moment**	1.37 (0.26)	1.00 (0.17)	0.0 (0.0)	0.95 (0.08)	0.17	0.10	1.38 (0.16)	1.00 (0.05)	0.0 (0.0)	0.97 (0.04)	0.08	0.08

### Kinetics

Regarding the intra-subject repeatability of the kinetics obtained with PiG (Table 1), the R^2^ was always greater than 0.91, and the standard deviation of the scaling factor was lower than 0.14 for all the examined variables. The average MAV was 0.22 Nm/kg for the hip, knee and ankle moments.

Table 2 shows the inter-operator LFM coefficients, MAV and MAV_OC_ averaged over both subjects for PiG. The R^2^ was higher than 0.92 for all the kinetics acquired at KUL, and higher than 0.81 for the kinetics acquired at OPBG. The standard deviation of the scaling factor was lower than 0.23. There was no difference between MAV and MAV_OC_, with the exception of the left knee moment at KUL (MAV = 0.06 Nm/kg and MAV_OC_ = 0.05 Nm/kg). For the HBM model (Table 3), the R^2^ was greater than 0.94 at both laboratories. The standard deviation of a_1_ was lower than 0.20. The only difference between MAV and MAV_OC_ was found for the ankle moment at OPBG (MAV = 0.08 Nm/kg and MAV_OC_ = 0.07 Nm/kg).

Table 4 shows the results of the inter-laboratory comparison for both models and both subjects. The R^2^ of hip, knee and ankle moments obtained with PiG were greater than 0.83. The maximum standard deviation of the scaling factor was 0.22. The higher values of MAV and MAV_OC_ were obtained for the left ankle moment (MAV = 0.17 Nm/kg and MAV_OC_ = 0.10 Nm/kg), other MAVs were in mean equal to 0.20 Nm/kg. Concerning the HBM, the R^2^ was greater than 0.91, the maximum standard deviation of the scaling factor was of 0.19. The averaged MAV value was 0.06 Nm/kg.

### Electromyography

Table 5 shows the intra-subject analysis results, with averaged R^2^ ranging between 0.55 and 0.83 for all muscles. The standard deviation of the scaling factor was lower than 0.61 for all the variables.

**Table 5.** Intra-subject repeatability: mean and standard deviation values of Linear Fitting Method (LFM) coefficients for the EMG signals.

		LFM		
		a_1_	a_0_ [μV]	R^2^
Right	**Gluteus Medius**	1.00 (0.32)	0.00 (27.80)	0.70 (0.14)
	**Vastus Lateralis**	1.00 (0.20)	0.00 (3.28)	0.83 (0.07)
	**Rectus Femoris**	1.00 (0.35)	0.00 (2.16)	0.63 (0.11)
	**Medial Hamstring**	1.00 (0.40)	0.00 (3.00)	0.68 (0.16)
	**Biceps Femoris**	1.00 (0.61)	0.00 (6.36)	0.58 (0.17)
	**Gastrocnemius**	1.00 (0.10)	0.00 (3.52)	0.74 (0.08)
	**Anterior Tibialis**	1.00 (0.23)	0.00 (7.55)	0.67 (0.08)
	**Soleus**	1.00 (0.22)	0.00 (4.68)	0.65 (0.12)
Left	**Gluteus Medius**	1.00 (0.27)	0.00 (2.58)	0.83 (0.05)
	**Vastus Lateralis**	1.00 (0.17)	0.00 (3.47)	0.84 (0.07)
	**Rectus Femoris**	1.00 (0.47)	0.00 (3.23)	0.57 (0.22)
	**Medial Hamstring**	1.00 (0.35)	0.00 (2.86)	0.56 (0.15)
	**Biceps Femoris**	1.00 (0.34)	0.00 (4.75)	0.55 (0.19)
	**Gastrocnemius**	1.00 (0.16)	0.00 (7.51)	0.73 (0.07)
	**Anterior Tibialis**	1.00 (0.18)	0.00 (8.01)	0.70 (0.07)
	**Soleus**	1.00 (0.23)	0.00 (5.51)	0.58 (0.11)

From an overall exam of the inter-operator comparison (Table 6), we concluded that the R^2^ ranged from 0.55 to 0.84 at KUL, and ranged from 0.49 to 0.82 at OPBG. When R^2^ ≤0.5, which occurred for left Medial Hamstring at OPBG, the other LFM coefficients were considered meaningless. The highest standard deviation of the scaling factor was found for the Biceps Femoris at KUL (0.46) and for the Medial Hamstring at OPBG (0.49).

**Table 6.** Inter-operator reproducibility: mean and standard deviation values of Linear Fitting Method (LFM) coefficients for the EMG signals of both subjects at KUL and OPBG labs.

		KUL			OPBG		
		LFM			LFM		
		a_1_	a_0_ [μV]	R^2^	a_1_	a_0_ [μV]	R^2^
Right	**Gluteus Medius**	1.00 (0.31)	0.00 (7.27)	0.78 (0.15)	1.00 (0.78)	0.00 (0.02)	0.73 (0.08)
	**Vastus Lateralis**	1.00 (0.18)	0.00 (2.67)	0.84 (0.07)	1.00 (0.29)	0.00 (4.48)	0.76 (0.08)
	**Rectus Femoris**	1.00 (0.39)	0.00 (2.64)	0.59 (0.15)	1.00 (0.32)	0.00 (1.79)	0.55 (0.13)
	**Medial Hamstring**	1.00 (0.40)	0.00 (3.11)	0.66 (0.15)	0.98 (0.20)	0.00 (2.54)	0.64 (0.10)
	**Biceps Femoris**	1.00 (0.46)	0.00 (5.19)	0.66 (0.16)	1.00 (0.33)	0.00 (8.11)	0.58 (0.13)
	**Gastrocnemius**	1.00 (0.21)	0.00 (4.83)	0.73 (0.17)	1.00 (0.26)	0.00 (4.22)	0.68 (0.11)
	**Anterior Tibialis**	1.00 (0.24)	0.00 (7.38)	0.68 (0.08)	1.00 (0.23)	0.00 (5.02)	0.63 (0.15)
	**Soleus**	1.00 (0.21)	0.00 (4.24)	0.65 (0.11)	1.00 (0.24)	0.00 (3.55)	0.67 (0.08)
Left	**Gluteus Medius**	1.00 (0.24)	0.00 (3.68)	0.79 (0.08)	1.00 (0.32)	0.00 (4.42)	0.82 (0.07)
	**Vastus Lateralis**	1.00 (0.17)	0.00 (1.50)	0.77 (0.07)	1.00 (0.34)	0.00 (5.16)	0.78 (0.12)
	**Rectus Femoris**	1.00 (0.42)	0.00 (2.62)	0.55 (0.15)	1.00 (0.40)	0.00 (3.80)	0.60 (0.17)
	**Medial Hamstring**	1.00 (0.36)	0.00 (2.05)	0.51 (0.13)	1.00 (0.49)	0.00 (3.21)	0.49 (0.18)
	**Biceps Femoris**	1.00 (0.31)	0.00 (3.35)	0.61 (0.17)	1.00 (0.30)	0.00 (9.02)	0.65 (0.16)
	**Gastrocnemius**	1.00 (0.34)	0.00 (7.35)	0.74 (0.10)	1.00 (0.17)	0.00 (6.72)	0.72 (0.08)
	**Anterior Tibialis**	1.00 (0.18)	0.00 (6.35)	0.69 (0.08)	1.00 (0.29)	0.00 (3.00)	0.70 (0.10)
	**Soleus**	1.00 (0.23)	0.00 (5.61)	0.67 (0.07)	1.00 (0.43)	0.00 (8.83)	0.56 (0.14)

The inter-laboratory results are reported in Table 7. R^2^ values were, in general, lower than those obtained for the inter-operator analysis and ranged between 0.44 and 0.77. The muscle activations that showed R^2^ ≤0.5 were the right and left Rectus Femoris, and the left Medial Hamstring. The highest standard deviation of the scaling factor was found (0.54) for the left Rectus Femoris.

**Table 7.** Inter-laboratory reproducibility: mean and standard deviation values of Linear Fitting Method (LFM) coefficients for the EMG signals.

		LFM		
		a_1_	a_0_ [μV]	R^2^
Right	**Gluteus Medius**	1.00 (0.14)	0.00 (11.60)	0.71 (0.03)
	**Vastus Lateralis**	1.00 (0.31)	0.00 (4.33)	0.73 (0.09)
	**Rectus Femoris**	1.00 (0.43)	0.00 (3.34)	0.49 (0.18)
	**Medial Hamstring**	1.00 (0.36)	0.00 (2.82)	0.61 (0.14)
	**Biceps Femoris**	1.00 (0.41)	0.00 (10.70)	0.58 (0.16)
	**Gastrocnemius**	1.00 (0.33)	0.00 (5.90)	0.67 (0.10)
	**Anterior Tibialis**	1.00 (0.27)	0.00 (11.07)	0.58 (0.12)
	**Soleus**	1.00 (0.26)	0.00 (4.21)	0.62 (0.10)
Left	**Gluteus Medius**	1.00 (0.30)	0.00 (4.41)	0.77 (0.08)
	**Vastus Lateralis**	1.00 (0.41)	0.00 (3.81)	0.72 (0.11)
	**Rectus Femoris**	1.00 (0.54)	0.00 (5.08)	0.49 (0.17)
	**Medial Hamstring**	1.00 (0.52)	0.00 (3.97)	0.44 (0.17)
	**Biceps Femoris**	1.00 (0.52)	0.00 (7.37)	0.55 (0.19)
	**Gastrocnemius**	1.00 (0.33)	0.00 (8.88)	0.71 (0.09)
	**Anterior Tibialis**	1.00 (0.26)	0.00 (7.93)	0.67 (0.09)
	**Soleus**	1.00 (0.30)	0.00 (7.31)	0.58 (0.11)

## Discussion

This study aimed to evaluate the intra-subject repeatability, the inter-operator and the inter-laboratory reproducibility of kinematics, kinetics and EMG data obtained from two typically developing children, accounting for the differences of two biomechanical models.

As regards the intra-subject repeatability, the study of the correlations obtained for the sagittal kinematics highlighted the pelvis as the least repeatable segment. This could be due to the noise related to segment with limited range of motion (ROM) using a marker-based approach (Kadaba et al. 1989). The ankle showed lower values of R^2^ than those of hip and knee for both models, which is most likely due to the smaller joint ROM (Røislien et al. 2012). The MAV increased with the increasing of the joint ROM (Di Marco et al. 2018); in particular, the intra-subject variability in the sagittal plane never exceeded the 10% of the ROM for all the kinematics. The only exception was observed for the pelvic tilt, which displayed MAV values equal to the 90% of its ROM in the worst case. Considering the coronal and transverse planes, high averaged values of R^2^ and low standard deviation values of a_0_ and a_1_ were found, indicating a strong similarity in the waveform patterns and high repeatability among data.

Similar to the intra-subject comparison, the coefficients a_1_ and a_0_ for the inter-operator reproducibility of hip, knee and ankle curves related both to PiG and HBM models on the sagittal plane showed high similarity among the curve patterns, and the averaged determination coefficients were close to 1 with a standard deviation that never exceed 0.10. The difference between MAV and MAV_OC_ was negligible in most cases, suggesting a low offset among the curves obtained from the sagittal data collected by the two operators at each lab. Slightly higher variabilities were found, instead, for the frontal and transverse kinematics with respect to the intra-subject variabilities. The previously indicated outcomes could be ascribed to the lower effects of marker misplacements of the kinematics in the sagittal plane, that is the direction of movement progression, with the highest ROM values of angular joints (Kadaba et al. 1989). Moreover, the flexion/extension axis is the first rotation axis of the joint coordinate system, and a proper alignment of that axis is crucial and might affect the estimated rotations in the frontal and transverse planes. Therefore, marker misplacements can imply a misalignment of the flexion/extension axis with the anatomical one, generating cross-talk between the motion on the sagittal plane and on the other ones (Ferrari et al. 2008). This cross-talk was clearly observed at the right knee and ankle transversal rotations evaluated at OPBG according to PiG where the differences between MAV and MAV_OC_ were equal to 30% of their respective ROM. MAV values calculated for knee and ankle rotations of both subjects at OPBG were clearly higher than those obtained for the left side evaluated at the same lab, and for both sides at KUL; whereas MAV_OC_ were comparable for both subjects at both laboratories (Table 1 and 2). Hence, from a comparative analysis of MAV and MAV_OC_, the knee and ankle rotations were most affected by a potential misplacement of the marker on the shank, which is the marker in common to both segments according to PiG. This is also confirmed by the literature (Davis et al. 1991; Cappozzo et al. 2005; Leardini et al. 2005), where it was reported that the marker on the shank should be considered as the main responsible for the cross-talk on the transverse plane. The placement of the shank marker appears to be fundamental for the measurements of ankle and knee rotations, even though the knee internal-external rotation is not commonly considered in the clinical routine due to its low precision (Ramsey and Wretenberg 1999). The inter-operator reproducibility of hip and knee joint moments was lower than the ankle moment. This may be explained by the inherent uncertainties in the estimation of lower limb joint centers (Kadaba et al. 1989) and, therefore, the propagation of the aforementioned uncertainties along the biomechanical chain of the lower limbs may have caused cumulative effects in the estimation of the moments moving from the ankle to the knee and hip joints.

As regards the inter-laboratory comparison, the kinematic parameters evaluated in frontal and transverse planes are more different rather than the ones evaluated in the sagittal plane, confirming the outcomes of the inter-operator comparisons. The differences between MAV and MAV_OC_, observed in the knee and ankle transversal rotations plane, were considerably higher than those resulting from the inter-operator comparison, showing an offset that reached the 50% of their ROM. This finding is in accordance with Gorton et al. (2009). In fact, the offset increments are linked to the marker misplacements, which may be high when two operators perform the marker positioning and are reasonably expected to be higher when the two operators come from two different laboratories. The reproducibility of pelvic tilt dramatically worsened in the inter-laboratory comparison: the R^2^ were lower than 0.45 and the MAV values reached 150% of the ROM. This is due to the high variability of pelvic tilt observed for the intra-subject analysis, as it leads to higher differences when comparing curves among subjects collected by different operators in different centers.

For the two adopted biomechanical models, a direct comparison of kinematics and kinetics by means of the proposed similarity indices is not possible due to the different definitions adopted for segments and joints (Di Marco et al. 2016b). From a qualitative comparison of the results, HBM seemed to yield more reproducible outcomes than PiG. This may be due to the higher number of joint constraints used in HBM (i.e. a hinged knee joint and two hinges in the ankle joint). However, it is worth considering that the PiG model also evaluates knee ab/adduction and rotation as model outputs, which yielded the lower correlation values, whereas these variables are not calculated in HBM. Indeed, the values of inter-operator and inter-laboratory reproducibility of the kinematics and kinetics were found to be comparable between models. As an overall observation on PiG and HBM, a higher reproducibility was found for kinematics and kinetics in the sagittal plane, whereas a lower reproducibility was found for the coronal and transverse rotations, confirming the findings obtained in (Ferrari et al. 2008).

The EMG signals showed lower intra-subject repeatability, inter-operator and inter-laboratory reproducibility than the kinematic and kinetic data, confirming the findings of Kadaba et al. (1989). In fact, the determination coefficients extracted from EMG data never exceeded 0.85. Although EMG curves showed similar patterns, they were affected by a delay between the activation peaks of the muscle signals (Figure 3), probably due to the high stride to stride variability inherent to the EMG data. Thus, the observed lower values of the determination coefficient R^2^ are most likely ascribed to the time shift between the curves, which invalidated the hypothesis of linearity relationship between the curves (Di Marco et al. 2018). Moreover, the EMG intra-subject repeatability and inter-operator reproducibility were comparable, suggesting that the physiological muscle activation variability predominates on the difficulty of accurately placing surface electrodes, which is also particularly challenging in the pediatric population (Granata et al. 2005).

The inter-laboratory reproducibility in EMG signals was only slightly worse than the inter-operator one. This is not surprising as the two centers used the same instrumentation, selected the same electrode-placement protocol, and adopted an identical post-processing procedure. Our findings confirmed the results reported by Kleissen et al. (1997), who found a clear similarity in the EMG waveform patterns among laboratories.

## Conclusions

High levels of repeatability and reproducibility were found for the sagittal kinematic and kinetic variables, while it was lower for EMG data. The pelvis segment proves to be the least repeatable segment and therefore it must be interpreted with care. The inter-operator variability gives a relevant contribution to the overall reproducibility of kinematic and kinetic gait data since it led to higher differences when comparing curves estimated from data collected in different centers. This is mainly due to the offset generated by the different marker positioning performed by different operators. In particular, the angles evaluated in the frontal and transverse planes should be carefully interpreted since they are most sensitive to the different marker positioning and they can varied as the operator and the laboratory varied. Repeatability and reproducibility were in general better for HBM than for PIG. The main contribution for the variability of the EMG signals can be ascribed to the intra-subject variability and, consequently, to the physiological stride to stride muscle activation variability. In conclusion, the variability of gait analysis measurements due to different operators or laboratories can be neglected, as long as experienced operators collect the data following well-defined and standardized experimental and post-processing procedures.
